# Priority Effects in the Apple Flower Determine If the Siderophore Desferrioxamine Is a Virulence Factor for Erwinia amylovora CFBP1430

**DOI:** 10.1128/aem.02433-21

**Published:** 2022-03-14

**Authors:** Laurin Müller, Denise C. Müller, Sandrine Kammerecker, Marco Fluri, Lukas Neutsch, Mitja Remus Emsermann, Cosima Pelludat

**Affiliations:** a Plant Pathology and Zoology in fruit and vegetable production, Agroscope, Waedenswil, Switzerland; b Centre for Food Safety and Quality Management, ZHAW School of Life Sciences and Facility Management, Waedenswil, Switzerland; c Research Group for Food Microbiology, ZHAW School of Life Sciences and Facility Management, Waedenswil, Switzerland; d Bioprocess Technology, ZHAW School of Life Sciences and Facility Management, Waedenswil, Switzerland; e Institute of Biology-Microbiology, Freie Universität Berlin, Berlin, Germany; f Virology, Bacteriology and Phytoplasmology, Agroscope, Nyon, Switzerland; University of Tennessee at Knoxville

**Keywords:** *Erwinia amylovora*, desferrioxamine, siderophore mutants, apple flowers, iron deficiency, replication, secondary colonization

## Abstract

Iron is crucial for bacterial growth and virulence. Under iron-deficiency bacteria produce siderophores, iron chelators that facilitate the iron uptake into the cell via specific receptors. Erwinia amylovora, the causative agent of fire blight, produces hydroxamate-type desferrioxamine siderophores (DFO). The presented study reassesses the impact of DFO as a virulence factor of E. amylovora during its epiphytic phase on the apple flower. When inoculated in semisterile Golden Delicious flowers no difference in replication and induction of calyx necrosis could be observed between E. amylovora CFBP1430 siderophore synthesis (DfoA) or uptake (FoxR receptor) mutants and the parental strain. In addition, mutant strains only weakly induced a *foxR* promoter-*gfp*mut2 reporter construct in the flowers. When analyzing the replication of the receptor mutant in apple flowers harboring an established microbiome, either naturally, in case of orchard flowers, or by pre-inoculation of semisterile greenhouse flowers, it became evident that the mutant strain had a significantly reduced replication compared to the parental strain. The results suggest that apple flowers *per se* are not an iron-limiting environment for E. amylovora and that DFO is an important competition factor for the pathogen in precolonized flowers.

**IMPORTANCE** Desferrioxamine is a siderophore produced by the fire blight pathogen E. amylovora under iron-limited conditions. In the present study, no or only weak induction of an iron-regulated promoter-GFP reporter was observed on semisterile apple flowers, and siderophore synthesis or uptake (receptor) mutants exhibited colonization of the flower and necrosis induction at parental levels. Reduced replication of the receptor mutant was observed when the flowers were precolonized by microorganisms. The results indicate that apple flowers are an iron-limited environment for E. amylovora only if precolonization with microorganisms leads to iron competition. This is an important insight for the timing of biocontrol treatments.

## INTRODUCTION

**FIG 1:**
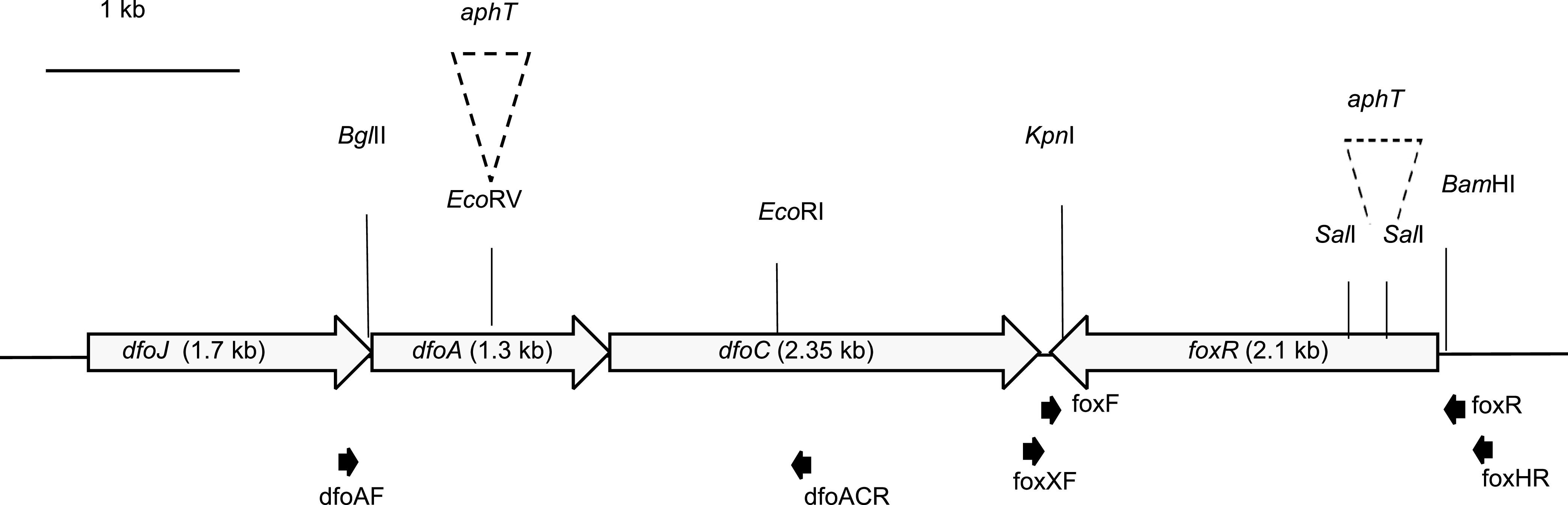
Schematic representation of the ferrioxamine cluster in E. amylovora CFBP1430. Large gray arrows indicate coding sequences. Small black arrows show the position and direction of primers used for site-directed mutagenesis of the *dfoA* and *foxR* genes. A kanamycin resistance cassette (*aphT*) was inserted into the EcoRV cutting site of the *dfoA* gene, resulting in the desferrioxamine synthesis mutant EAdfoA. Replacing the 203 pb SalI fragment of the *foxR* gene by *aphT* resulted in the ferrioxamine receptor mutant EAfoxR.

Even though iron is the fourth most abundant element on Earth, under oxidative conditions its bioavailability is limited to microorganisms. This limitation is due to the reduction of Fe^2+^ to the insoluble Fe^3+^ state. As iron is an essential cofactor in reactions such as DNA replication or protection against oxygenated radicals, many microorganisms have evolved high-affinity systems to acquire iron, so-called siderophores. Siderophores are molecules with low molecular masses (200 - 2000 Da) that are produced by bacteria when the intracellular iron concentration is low (1, 2). Siderophores chelate Fe^3+^ with a very high affinity and the chelated complexes are recognized by highly selective outer-membrane TonB-dependent receptors on the cell surface of Gram-negative bacteria. The receptors bind the Fe^3+^ siderophore complex, which is then actively transported across the membrane through an energy-dependent system into the cytosol, where the iron is released (3). The regulation of iron metabolism in bacteria is mediated by the ferric-uptake regulator protein (Fur). Fur represses transcription of iron-uptake associated genes upon interaction with its corepressor Fe^2+^ by binding the consensus sequence known as “Fur box” within Fur-regulated promoters (4, 5). The fire blight pathogen Erwinia amylovora is considered to be the most important threat to pome fruit production (e.g., apple, pear, and quince) globally. The pathogen is vectored by flower-foraging insects, mainly bees (6, 7). It infects the host tissue through the nectarthodes after an epiphytic phase during which it replicates on the flower stigma. E. amylovora produces hydroxamate-type desferrioxamine siderophores (DFOs), mostly DFO E (nocardamine) (8, 9). DFO E is encoded by the *dfoJAC* gene cluster, the specific receptor for the passage of the ferric complex across the outer membrane by *foxR*. The *foxR* gene is located on the complementary strand directly downstream of *dfoC* (Fig. 1). Both *foxR* and the *dfoJAC* cluster are regulated by Fur via their Fur box containing promoters (10, 11).

For E. amylovora CFBP1430 a dual function of DFO has been proposed. The siderophore plays a role in cell protection against the host oxidative burst elicited during infection and is critical for its iron acquisition (12). Transposon mutants either defective in the DFO biosynthetic pathway (*dfoA* mutant) or uptake of the ferric complex (*foxR* mutant) have been studied. Whereas a *dfoA* mutation disrupts the synthesis of the siderophore, a mutation in the FoxR receptor leads to an accumulation of the siderophore in the external medium due to the lack of reuptake (10, 12). When tested on apple flowers the *dfoA* and *foxR* mutants revealed a reduction in their growth (2 orders of magnitude) and in their ability to initiate necrotic symptoms compared to the wild-type strain (12).

Studies on a pyoverdine siderophore negative mutant of Pseudomonas orientalis F9 which inhibited growth of E. amylovora as successful as the parental strain (13) prompted us to reassess the impact of DFO as a virulence factor of E. amylovora during the epiphytic phase in the apple flower. To this end siderophore synthesis (*dfoA*) and uptake (*foxR*) mutant strains were tested on various Golden Delicious (GD) flowers for their growth rates, calyx necrosis, and induction of a *foxR* promoter-*gfp*mut2 reporter construct.

In semisterile GD flowers siderophore mutants have no disadvantage in terms of replication and necrosis induction compared to the parental strain, nor does E. amylovora CFBP1430 induce *foxR* when colonizing the stigma. Significant growth deficiency of a receptor mutant was observed in pre-colonized flowers, either by a naturally evolved microbiome in the orchard or by pre-incubation of a competitor on semisterile greenhouse flowers. It has been shown that precolonization of semi sterile leaves can drastically decrease the reproductive success of secondary colonizers (14). The present study shows how the priority effects of colonization alter the context-specific dependence of a pathogenicity factor during flower colonization.

The results indicate that the DFO system of E. amylovora is required when the pathogen performs as a secondary flower colonizer.

## RESULTS

### Siderophore synthesis and growth of parental and mutant strains.

**FIG 2:**
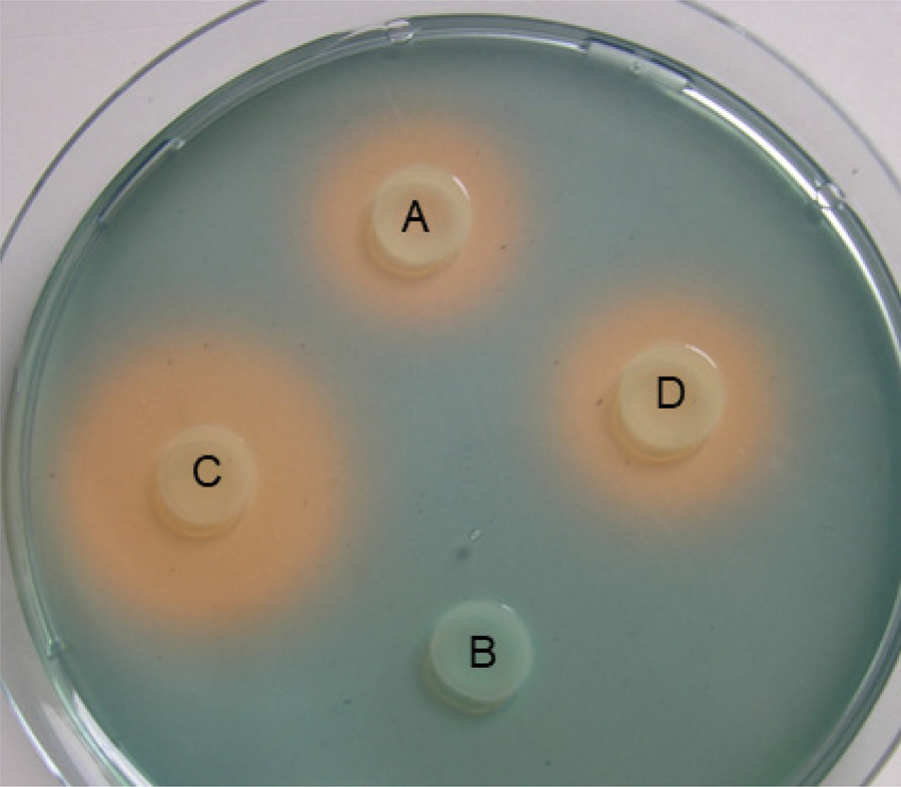
Siderophore indication CAS-agar with E. amylovora CFBP1430 parental strain EA^S^ (A), desferrioxamine synthesis mutant EAdfoA (B), ferrioxamine receptor mutant EAfoxR (C) and complemented receptor mutant EAfoxRco (D). The orange halos are indicative for siderophore production.

Parental strain EA^S^ (E. amylovora CFBP1430 Sm^R^, Table 1), corresponding desferrioxamine synthesis mutant E. amylovora EAdfoA, ferrioxamine receptor mutant EAfoxR and complemented mutant EAfoxRco were analyzed on CAS detection agar for siderophore synthesis (Fig. 1, Fig. 2). As expected, EAdfoA was not able to produce an orange halo indicative for siderophore synthesis. The receptor mutant EAfoxR produced a larger halo than the parental strain EA^S^ due to its inability to take up the siderophore-iron complex and hence inability to repress the expression of siderophore production. After complementation of EAfoxR with the plasmid pEA*foxR* (EAfoxRco) the halo size was reduced.

**TABLE 1:** Strains used in this study

Strain	Genotype and/or phenotype	Reference
Escherichia coli DH5α	*endA1 hsdR17* (r_K_^−^ m_K_^+^) *supE44 thi-1 recA1 gyrArelA1* Δ(*lacZYA-argF*)*U169* (80*lacZΔM15*)	41
E. coli S17-1λ	*pir^+^ tra^+^*	32
Erwinia amylovora CFBP1430	Isolated in 1972 from a *Crataegus* sp.	42
EA^S^: E. amylovora CFBP1430 Sm^R^	Spontaneous streptomycin resistant mutant of Erwinia amylovora CFBP1430	This study
EA^S^gfp: E. amylovora CFBP1430 Sm^R^, (p*EAfoxRgfp*mut2) Amp^R^	Spontaneous streptomycin resistant mutant of Erwinia amylovora CFBP1430, carrying reporter plasmid p*foxR* promoter-*gfp*mut2	This study
EAdfoA: E. amylovora CFBP1430*EAdfoA*::aphT Sm^R^, Kan^R^	Siderophore negative mutant of EA^S^, CAS negative	This study
EAdfoAgfp: E. amylovora CFBP1430*EAdfoA*::aphT (p*foxRgfp*mut2), Amp^R^, Sm^R^, Kan^R^	Siderophore negative mutant of EA^S^, carrying reporter plasmid p*foxR* promoter-*gfp*mut2	This study
EAfoxR: E. amylovora CFBP1430*foxR*::aphT, Sm^R^, Kan^R^	Siderophore receptor mutant of EA^S^, CAS halo oversized	This study
EAfoxRco: E. amylovora CFBP1430*EAfoxR*:: aphT, (pKS*foxR*),Amp^R^, Sm^R^, Kan^R^	Siderophore receptor mutant of EA^S^, partially complemented with plasmid pKS*foxR*	This study
EAfoxRgfp: E. amylovora CFBP1430*foxR*::aphT,(p*foxRgfp*mut2), Amp^R^, Sm^R^, Kan^R^	Siderophore receptor mutant of EA^S^ carrying reporter plasmid p*foxR* promoter-*gfp*mut2	This study
PW: Pantoea vagans C9-1W	Pantoea vagans C9-1 variant that Lacks the 530-kb megaplasmid pPag3. pPag3 encodes among other things (e.g., carbon utilization, thiamin, carotenoids) the desferrioxamine synthesis genes.	43
PWgfp: P. vagans C9-1W (p*foxRgfp*mut2), Amp^r^	Pantoea vagans C9-1W carrying reporter plasmid *foxR*promoter-*gfp*mut2	This study

**FIG 3:**
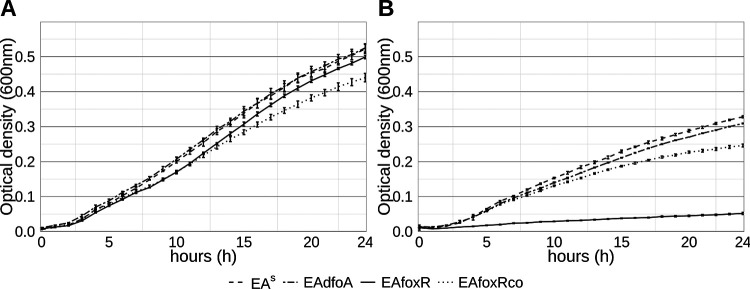
LB (A) and KB (B) growth curves of E. amylovora CFBP1430 strains EA^S^ (solid), desferrioxamine synthesis mutant EAdfoA (dotted), ferrioxamine receptor mutant EAfoxR (dashed) and complemented mutant EAfoxRco (dotdash). Error bars represent standard deviations.

While growing in LB medium, all strains grew similarly with the exception of the complemented strain EAfoxRco which showed slightly impaired growth (Fig. 3A). When inoculated in iron-limited KB medium, EAfoxR showed a significant growth reduction compared to the parental strain, which was partially restored in the complemented strain EAfoxRco (Fig. 3B).

### Induction of flower necrosis.

**FIG 4:**
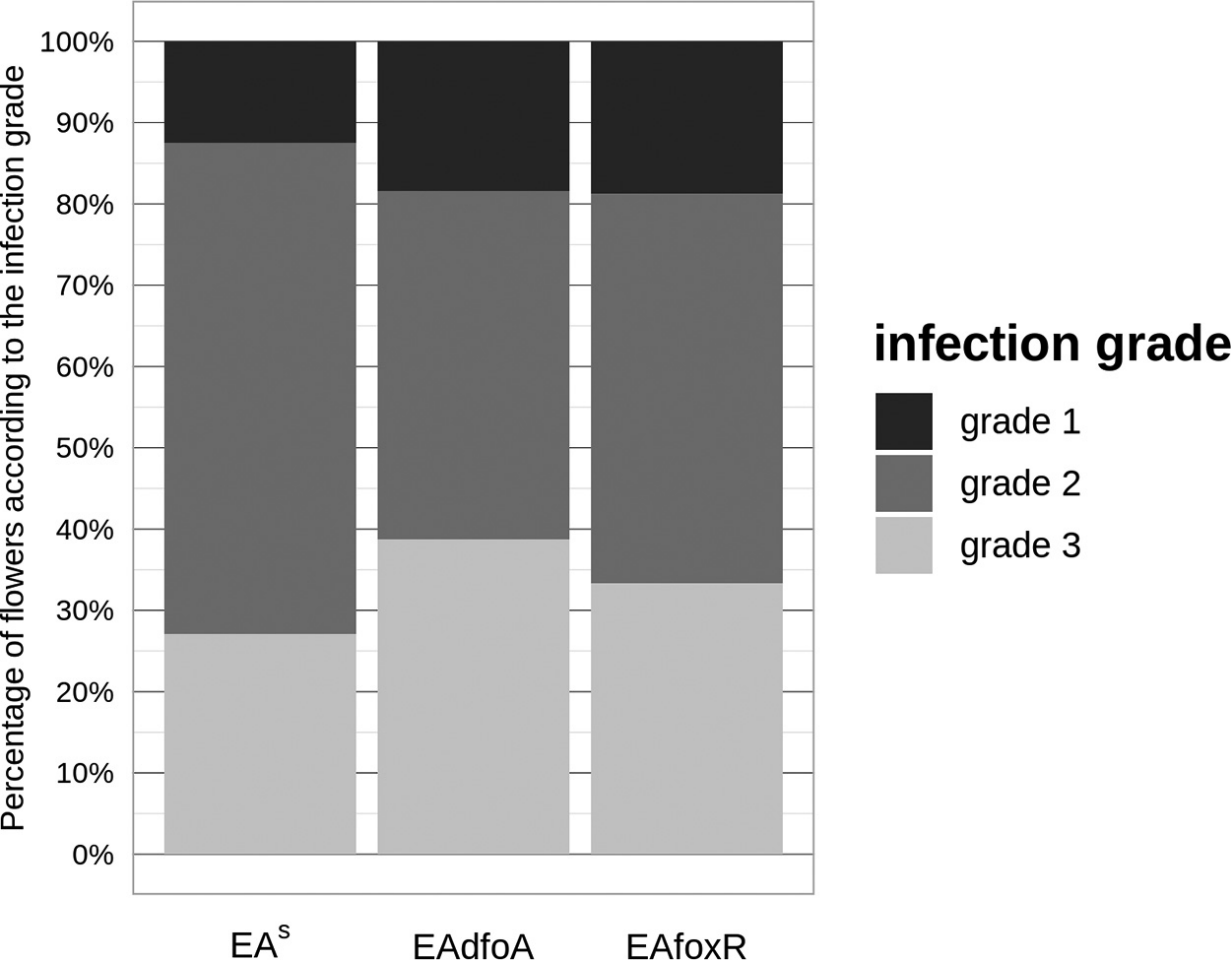
Percentage fraction of flowers in each infection grade. Each column represents 48 evaluated flowers from two independent detached flower assay experiments. Flowers were inoculated onto the hypanthium with E. amylovora CFBP1430 strains EA^S^, desferrioxamine synthesis mutant EAdfoA, and ferrioxamine receptor mutant EAfoxR. Evaluation of the flowers infection grade was performed after 4 days of incubation at 26°C according to the following scale: grade 1: calyx green; grade 2: calyx necrotic; grade 3: calyx and pedicel necrotic.

The ability of the strains to induce necrosis in apple flowers was tested on detached GD flowers. The infection grades (Fig. 4) and the resulting severity grades of parental strain EA^S^ (71.5), siderophore synthesis mutant EAdfoA (73.5), and receptor mutant EAfoxR (71.5) revealed no difference in the induction of flower necrosis.

### Bacterial densities on the stigma and hypanthium.

**FIG 5:**
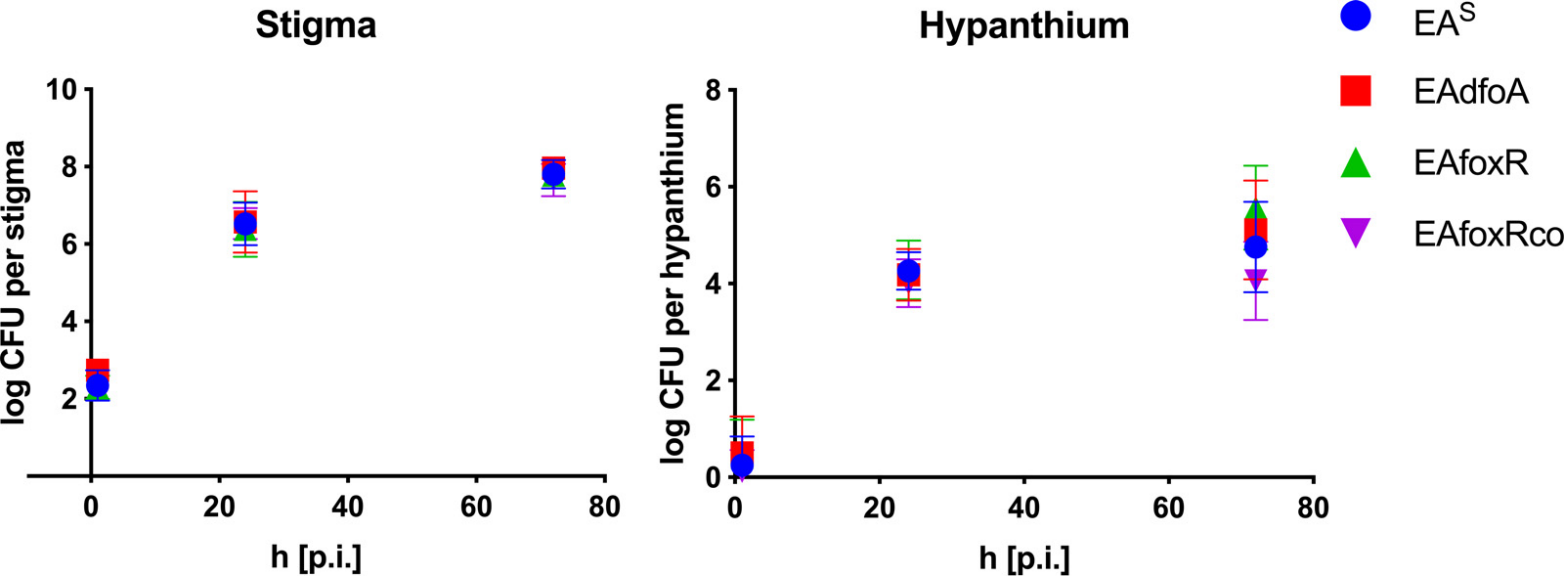
CFU of parental strain E. amylovora CFBP1430 EA^S^, corresponding desferrioxamine synthesis mutant (EAdfoA), ferrioxamine receptor mutant (EAfoxR), and complemented ferrioxamine receptor mutant (EAfoxRco) 0 h, 24 h and 72 h p.i. on stigma and hypanthium of detached GD flowers.

In the established assay for induction of flower necrosis as performed above, the inoculum is directly applied onto the hypanthium of the flowers. Therefore, the assay omitted the important step of the replication of the pathogen on the stigmata. To assess the impact of the mutations on the replication, stigmata of detached GD flowers were dipped into bacterial suspensions of EA^S^, EAdfoA, EAfoxR, and the complemented receptor mutant EAfoxRco. Parental and mutant strains replicated equally on stigmata and only the complemented EAfoxRco strain revealed a slightly reduced CFU on the hypanthium (Fig. 5).

### Bacterial densities and necrosis induction in flowers of GD trees.

**FIG 6:**
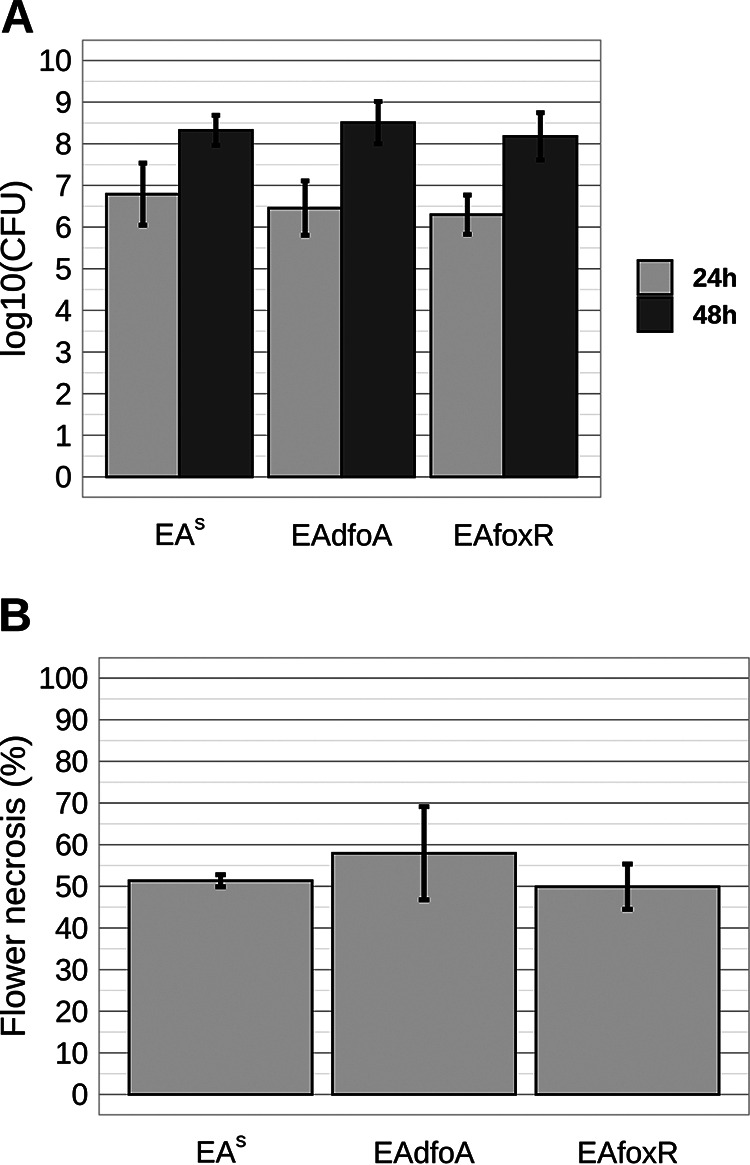
CFU and necrosis of GD flower calyxes after spray inoculation of flowering GD trees with E. amylovora CFBP1430 strains EA^S^, EAdfoA, and EAfoxR. (A) CFU were determined for 10 flowers per strain 24 h and 48 h p.i. Error bars represent the standard deviation from the mean. (B) Disease symptoms of flowers rated 6 days after inoculation. Error bars represent the standard deviation of three trees. In total 413, 474 and 333 flowers were evaluated for EA^S^, EAdfoA and EAfoxR, respectively.

Three flowering GD trees each were sprayed with bacterial suspensions of EA^S^, EAdfoA and EAfoxR. In accordance with the previous results, there were no differences in the recovered CFU. The same was true for the induction of flower necrosis (Fig. 6A and B).

### *FoxR* promoter activity on apple flower stigmata.

None of the *in vivo* assays performed revealed a difference between E. amylovora parental and siderophore mutant strains regarding their replication on GD flowers and induction of necrosis. To verify the need for the siderophore system during the epiphytic phase of the fire blight pathogen, the Fur regulated *foxR* promoter was fused to the *gfp*mut2 encoding gene and the resulting reporter plasmid (p*foxRgfp*mut2) transformed into the parental and both mutant strains resulting in EA^S^gfp, EAdfoAgfp, and EAfoxRgfp. To test the functionality of the reporter-construct EAfoxRgfp was inoculated in iron deficient KB medium. In addition to the E. amylovora strains, the white variant of the well-studied E. amylovora antagonist P. vagans C9-1, P. vagans C9-1W (PW), was included in the assay as fluorescent positive-control PWgfp. PW is deficient of the megaplasmid pPag3, which carries the desferrioxamine siderophore encoding genes in this strain (Table 1). GFP measurements showed an induction of the reporter construct in both strains (Fig. S1).

**FIG 7:**
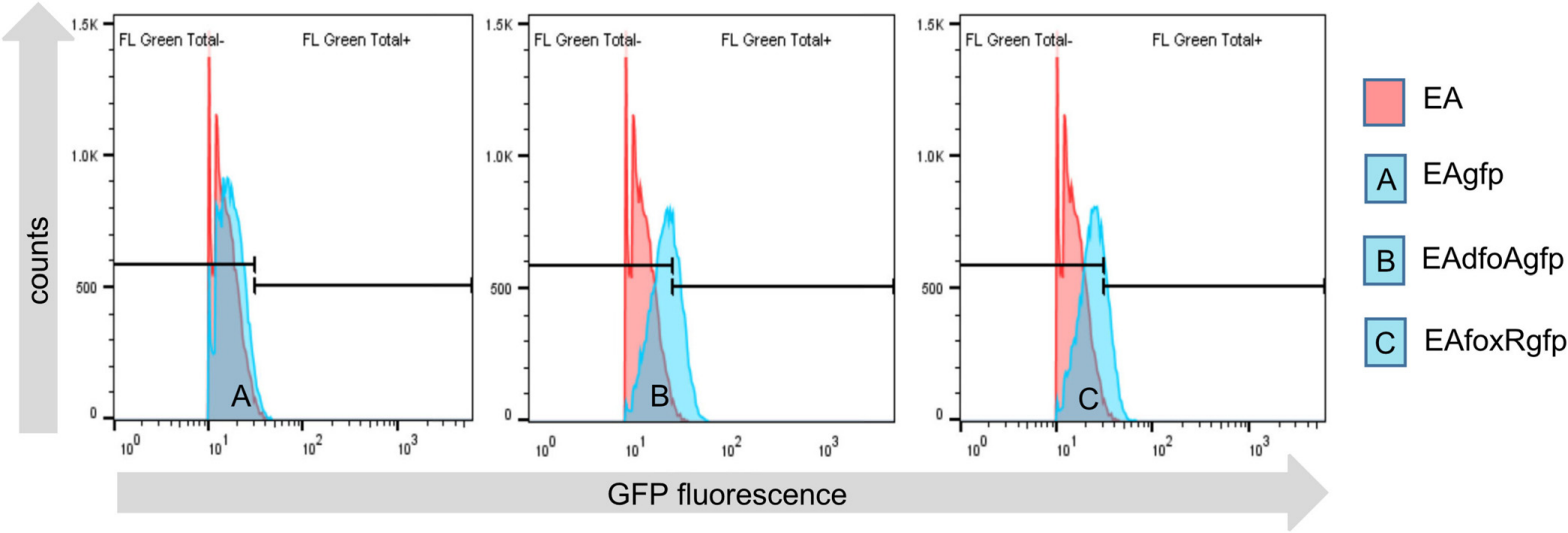
GFPmut2 expression under the control of the EA*foxR* promoter in E. amylovora CFBP1430 strains EA^S^ (EAgfp=A, blue), synthesis mutant EAdfoA (EAdfoAgfp=B, blue), and receptor mutant EAfoxR (EAfoxRgfp=C, blue) reisolated from GD flowers 48 h p.i. on the stigmata. E. amylovora CFBP1430, in red, lacking the reporter construct is the negative control.

When GFP measurements were performed *in planta*, none of the *Erwinia* strains tested showed a strong induction of the *foxR* promoter reporter (Fig. 7). The percentage of *gfp* induced cells was between 5% (EAfoxRgfp) to 10% (EAdfoAgfp), (Table 2). The parental strain showed no induction (0.4%). This is in contrast to control strain PWgfp, where 85% of the cells were GFP positive (Fig. S2), confirming the assay to be able to detect high GFP levels, if the reporter is induced *in planta*.

**TABLE 2:** Single cell GFP fluorescence of reisolated reporter strains 48 h p.i. from GD apple stigmata

Strain	Total count	Count GFP positive	Count GFP negative	% GFP positive
EA	21083	71	21012	0.3
EAgfp	22594	92	22502	0.4
EAdfoAgfp	21552	2214	19338	10.3
EAfoxRgfp	20149	962	19187	4.8

### Bacterial densities on the stigmata of greenhouse or orchard-grown GD flowers.

**FIG 8:**
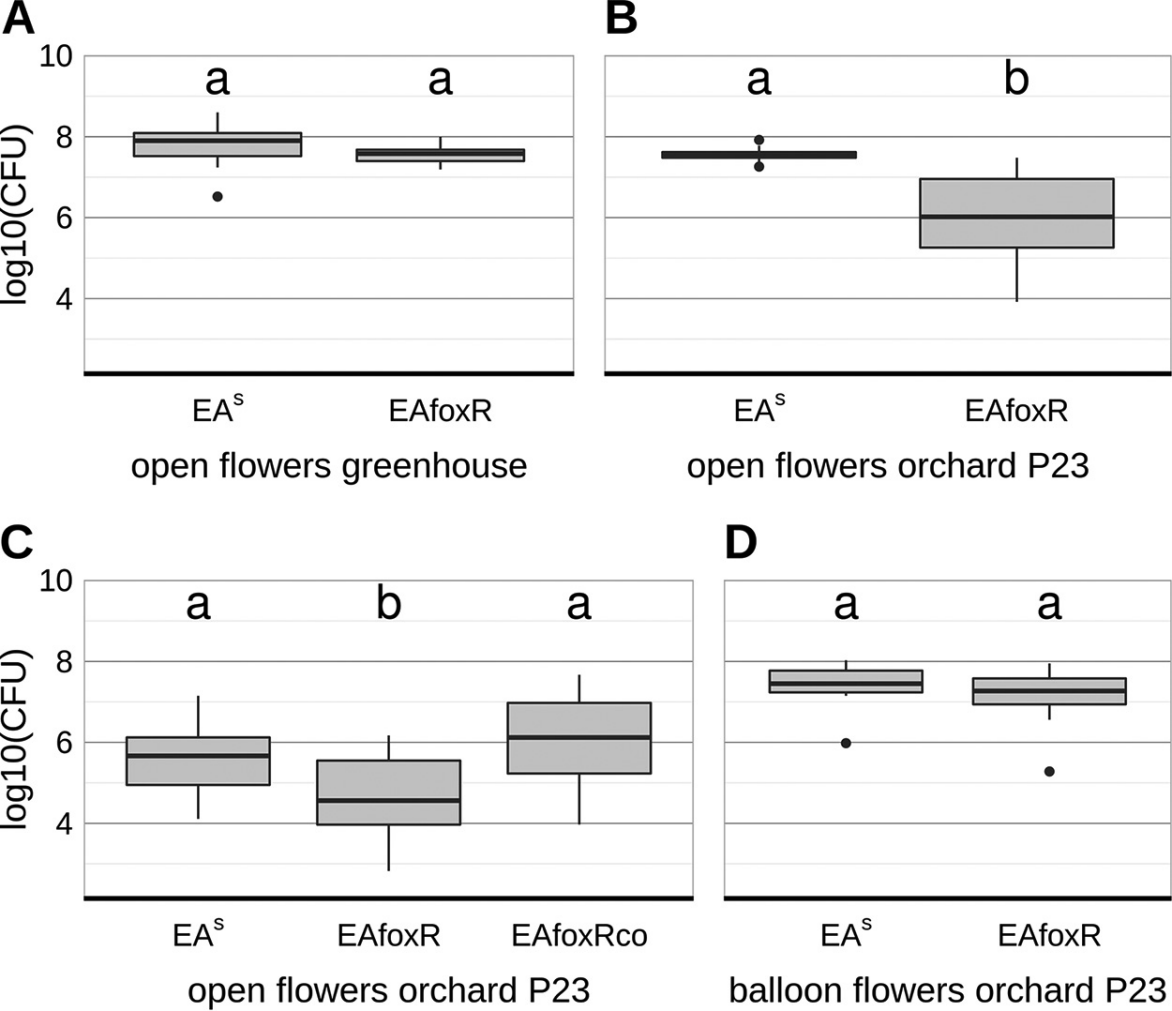
CFU of reisolated E. amylovora CFBP1430 strains 48 h p.i. (A) parental strain EA^S^ (*n* = 9) and mutant EAfoxR (*n* = 20) from semisterile GD flowers, greenhouse; (B) parental strain EA^S^ (*n* = 10) and mutant EAfoxR (*n* = 20) from open GD flowers, orchard-P23; (C) parental strain EA^S^ (*n* = 20), mutant EAfoxR (*n* = 20) and complemented mutant strain EAfoxRco (*n* = 19) from open GD flowers, orchard-P23; (D) parental strain EA^S^ (*n* = 10) and mutant EAfoxR (*n* = 9) from GD balloon flowers, orchard-P23. Error bars represent the standard deviation of the mean. Significant differences between treatments are marked with different letters (*P*-value < 0.05, one-way ANOVA, Tukey’s multiple-comparison test).

In contrast to the previous study (12) apple flowers from the above performed experiments originated from a closed compartment and were not exposed to nectar foraging insects, wind or rain. Thus, the observed difference is likely due to the colonization of the flowers with a microbiome that develops in an orchard environment but not in a closed compartment. To test this hypothesis, semisterile GD flowers from the greenhouse and open (microbiome bearing) flowers from the orchard-P23 were inoculated with EA^S^ or the EAfoxR receptor mutant. In semisterile flowers from the greenhouse both strains replicated equally (Fig. 8A). In accordance with the results of the previous study (12) the EAfoxR mutant revealed a reduced CFU in the orchard-P23 flowers compared to the parental strain (Fig. 8B). The complemented receptor mutant (EAfoxRco) multiplied as efficiently as the parental strain in microbiome containing orchard-P23 flowers (Fig. 8C, Fig. S3). When EA^S^ and EAfoxR were inoculated in freshly opened, semisterile balloon flowers from the orchard-P23, both strains multiplied equally well (Fig. 8D).

**FIG 9:**
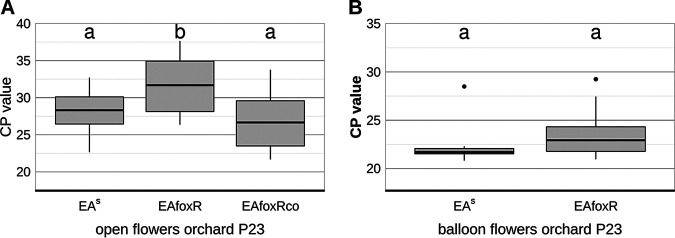
CP values of E. amylovora CFBP1430 strains. (A) EA^S^ (*n* = 20), corresponding mutant EAfoxR (*n* = 20) and complemented mutant strain EAfoxRco (*n* = 19) reisolated from open GD flowers, orchard-P23 after 48 h p.i.; (B) EA^S^ (*n* = 10) and mutant EAfoxR (*n* = 10) reisolated from GD balloon flowers, orchard-P23, 48 h p.i. The bacterial DNA was extracted from each infected flower and qPCR performed with an *ams*C (amylovoran synthesis) specific probe. Error bars represent the standard deviation of the mean. Significant differences between treatments are marked with different letters (*P*-value < 0.05, one-way ANOVA, Tukey’s multiple-comparison test).

To verify the CFU data gained by classical plating, an EA specific qPCR was performed on each of the flower samples from open- (EA^S^, EAfoxR, EAfoxRco) and balloon orchard-P23 flowers (EA^S^ and EAfoxR, Fig. 9), confirming the replication deficiency of the receptor mutant in open orchard flowers.

### Bacterial densities on the stigmata of pre-inoculated greenhouse GD flowers.

**FIG 10:**
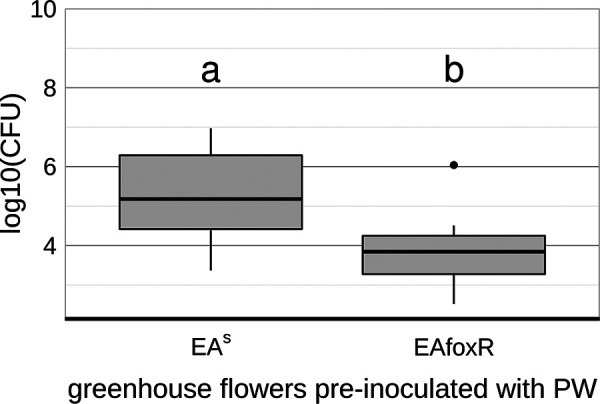
CFU of E. amylovora CFBP1430 strains EA^S^ (*n* = 20) and corresponding mutant EAfoxR (*n* = 20) 48 h p.i. onto PW pre-inoculated semisterile GD flowers from the greenhouse. Error bars represent the standard deviation of the mean. Significant differences between treatments are marked with different letters (*P*-value < 0.05, one-way ANOVA, Tukey’s multiple-comparison test).

To verify the impact of flower colonizers on the replication of receptor mutant and to exclude abiotic factors and changes in the nutrient composition of the open orchard flowers to be the main cause, freshly opened GD flowers from the greenhouse were pre-inoculated with PW as a competitor. Consistent with the results from open orchard-P23 GD flowers, EA^S^ was significantly better at colonizing the stigma than EAfoxR (Fig. 10). Performing the *ams*C qPCR using DNA templates consisting of 10 pooled samples confirmed the CFU data obtained (Fig. S4). The complemented strain EAfoxRco reached the parental CFU level in the pre-inoculated flowers (Fig. S5).

## DISCUSSION

The synthesis and regulation of siderophores, Fe^3+^ chelators (15), have been intensively studied in human-pathogenic bacteria, e.g., *Yersinia and* Salmonella enterica, demonstrating their role in mediating pathogen multiplication and development of virulence (16–18). The role of siderophores in plant microbial pathogenesis is less well studied. From Dickeya dadantii 3937, the causal agent of “soft rot,” it is known that the synthesis of siderophores is required for the systemic progression of maceration symptoms in the host (19) and Pseudomonas syringae pv. *tabaci* 6605 requires the siderophore pyoverdine for full virulence in tobacco (20). E. amylovora, the causal agent of fire blight produces desferrioxamine DFO siderophores, mainly DFO-E (8, 9). E. amylovora mutants defective in siderophore synthesis or uptake exhibited a reduced ability to colonize floral tissues and to cause necrosis, indicating that DFO is a virulence factor during the onset of infection (12). The presented study focuses on the DFO system and its impact on E. amylovora during the epiphytic state of the pathogen on the apple flower. To this end, a *dfoA* (EAdfoA) siderophore synthesis and a *foxR* (EAfoxR) receptor mutant were constructed. Neither the EAdfoA nor the EAfoxR mutant revealed a decrease in replication or in necrosis induction when applied onto GD flowers from a closed greenhouse. Additionally, a fluorescence whole-cell bio-reporter under the control of the Fur regulated *foxR* promoter was not induced in the parental strain and only weakly (5% to 10%) in the mutant strains when incubated on greenhouse-grown stigmata (Fig. 7). In contrast, in iron-limited KB medium, 93% (Fig. S1) of the receptor mutant cells were classified as GFP-positive.

These results imply that semisterile GD flowers do not represent an iron-limiting environment for E. amylovora and therefore, the lack of siderophore production or uptake is not a disadvantage in replication and necrosis induction for mutant strains. This is in contrast to P. fluorescens strain A506, an antagonist of E. amylovora, commercialized as BlightBan. An ice nucleation protein-based promoter reporter assay demonstrated that pyoverdine siderophore genes are upregulated on apple and pear flowers in growth conditions where blossoms were protected from rain and insect visitations, which indicates that those blossoms represented an iron-limited environment to P. fluorescens A506 (21).

The E. amylovora CFBP 1430 genome contains only remnants of the siderophore receptors ferrichrome (FhuA) and aerobactin (IutA) (22). The inactivation of such a potential source of Fe^3+^ acquisition possibly indicates that E. amylovora only requires siderophores for replication at some steps of its infection cycle, not necessarily the flower. It might be that E. amylovora compensates its iron need by additional systems present on its chromosome, e.g., SitABCD/YfeA-E that also can take up Fe^2+^ or the iron specific EfeUOB system (23–27).

The flowers used in these experiments originated from an enclosed facility and thus, were semisterile. Studies on the expression of *foxR* in inoculated apple leaves (28) already revealed differential expression of the gene depending on localization and bacterial density. Therefore, it is possible that the siderophore is critical for E. amylovora replication in the flower only if the pathogen competes with additional apple blossom colonizers. To test this hypothesis, open GD flowers from the orchard P23 were inoculated with the EA^S^ and the EAfoxR strain. The results confirmed a decreased ability of EAfoxR to replicate on precolonized flowers, which was reversed when the mutant strain was complemented (Fig. 8C and 9A). To verify that EAfoxR growth inhibition is due to competitors, freshly opened greenhouse-grown GD flowers were pre-inoculated with the strain PW. Similar to flowers grown in a natural environment, EAfoxR, in contrast to the complemented mutant, could not replicate to the same density as the parental strain.

The here presented results imply that iron is readily bioavailable to E. amylovora during its epiphytic phase on non-precolonized flowers. In already colonized flowers, either with a complex microbiome or a single competitor, EafoxR growth decreased to an extent that indicates the siderophore system as an important competitive factor in flower colonization. This underscores the importance of an initial flower colonization by epiphytes in pest management of E. amylovora.

## MATERIALS AND METHODS

### Cultivation of bacteria.

For apple flower inoculation and CFU determination after reisolation from infected flowers E. amylovora strains were grown at 26°C in Tryptic Soy broth (TSB, Oxoid) or on TSB plates (addition of 15 g liter**^−^**^1^ agar [Roth]). E. coli strains were cultivated at 37°C in Lysogeny broth (LB, Carl Roth) or on LB plates (addition of 15 g liter**^−^**^1^ agar). For growth curve experiments E. amylovora strains were grown in King's B medium (29) and in Lysogeny broth. Microorganisms used in the study are listed in Table 1. Where appropriate, medium was supplemented with kanamycin 40 mg liter^−1^, streptomycin 100 mg liter^−1^, rifampicin 100 mg liter^−1^, ampicillin 100 mg liter^−1^ or cycloheximide 100 mg liter^−1^, respectively.

### Construction of mutant strains.

To inactivate the *foxR* gene, a part of the gene was amplified using primers FoxF (5′-AAGCTAAACCAGCGATAAGTATAG-3′) and EAfoxR (5′-TCGTAACCGACGGTAGCCATC-3′) resulting in a 2.1 kb fragment harboring a BamHI and KpnI cutting site at the 5′ and 3′ prime end, respectively. The amplified fragment was digested with BamHI/KpnI and ligated into similarly digested plasmid pBluescript (pKS). Subsequently, the resulting vector was digested with SalI, which cuts twice within the open reading frame of the *foxR* gene, deleting 203 bp (Fig. 1). The excised region was replaced with a HindII cut kanamycin cassette from plasmid pSB315 (*aphT*), which lacks a transcriptional terminator (30). Using primers M13F (5′-CAGGAAACAGCTATGAC-3′) and M13R (5′-TGTAAAACGACGGCCAGT-3′) the *foxR*-kanamycin cassette was amplified, digested with BamHI/KpnI and ligated into the similarly digested suicide vector pKAS32 (31). The resulting construct, pSVEAfoxR was transformed into E. coli S17-1 (32, 33) and mobilized into a spontaneous streptomycin resistant mutant of E. amylovora CFBP1430 (EA^S^). Plasmid pKAS32 contains the *rpsL* gene encoding the S12 protein of the ribosomes, which is the target of streptomycin. Thereby insertion of the suicide vector into the chromosome results in a streptomycin sensitive phenotype of the formerly resistant strain, which counter selects double crossing over events. The receptor mutant EA^S^*foxR*::aphT (EAfoxR) was selected on TSB agar plates containing kanamycin and streptomycin. The insertion was confirmed by PCR and subsequent sequencing of the PCR product.

For complementation, the *foxR* gene and its native promoter were amplified using primers FoxHR (5′-AAAAAAAGCTTAACGTCCTCCTTCTTCTGGG-3′) and FoxXF (5′-GGGGGTCTAGAAAGCTAAACCAGCGATAAGTATAG-3′) which introduce a XbaI and HindIII cutting site, respectively (underlined). The resulting 2.6 kb PCR product was XbaI/HindIII digested and ligated into the similarly digested pKS vector. The resulting plasmid pKS*foxR* was electroporated into EAfoxR to generate the complemented mutant EAfoxRco.

To inactivate the *dfoA* gene, a 2.3 kb fragment of the gene was amplified using primers dfoA2661F (5′-CTGCTGCACAACGTGCTGCT-3′) and dfoA4940R (5′-GTGCATTACGGCCGTGAACA-3′) harboring an EcoRI and a BglII cutting site at the 5′ and 3′ end of the PCR product, respectively. The PCR product was EcoRI/BglIII digested and ligated into the similarly digested suicide vector pKAS32 and transformed into E. coli S17-1. The plasmid was EcoRV digested which cuts within the *dfoA* gene and the HindII cut kanamycin cassette from plasmid pSB315 was inserted. The resulting plasmid pSVdfoA was subsequently mobilized into EA^S^. EA^S^*dfoA*::aphT (EAdfoA) mutants were selected on KB agar plates containing kanamycin and streptomycin and confirmed by PCR.

### Construction of the *foxR* promoter-GFPmut2 construct.

To construct a *foxR* promoter-GFP reporter construct, 365 bp upstream up the *foxR* gene (including the upstream regulatory Fur sequences) were amplified using primers Pfox_XF (5′GGGGGTCTAGAAAGCTAAACCAGCGATAAGTATAG3′) and Pfox_PR (5′GGGGGCTGCAGGTTTATTCCCTTACAAAGATTA3′) with designed restriction sites XbaI and PstI (underlined). The resulting PCR product was XbaI/PstI digested and ligated into similarly digested plasmid pM965 (34) which places the GFPmut2 encoding gene in pM965 under the control of the *foxR* promoter. The resulting plasmid p*foxRgfp*mut2 was electroporated into the E. amylovora parental and mutant strains and P. vagans C9-1W.

### Siderophore indicator test.

Siderophore production was tested on Chrome azurol S (CAS) agar (35). Freshly grown colonies of EA^S^, EAdfoA, EAfoxR, and EAfoxRco grown on TSB agar plates with the appropriate antibiotics overnight were resuspended in 1× PBS buffer (NaCl 8 g liter^−1^, KCl 0.2 g liter^−1^, Na_2_HPO_4_·12H_2_O 2.9 g liter^−1^, KH_2_PO_4,_ 0.2 g liter^−1^, pH 7.2) to an OD_600nm_ = 1. Five μL of the resuspended cultures were applied onto the indicator agar. Formation of an orange halo around applied bacteria was indicative for siderophore production.

### Growth curves.

Bacterial growth was monitored using a Bioscreen C (Oy Growth Curves Ab Ltd., Helsinki). To study the effect of iron deficiency on EA^S^ and corresponding mutants, growth was observed in LB and iron-limited KB medium. Seven hundred μL of the respective media were inoculated with 3 μL of an overnight culture and loaded in three replicates (200 μL per well) on a Bioscreen C honeycomb plate. For each replicate 200 μL of the corresponding medium were used as a negative control. Measurement of optical density at 600 nm (OD_600nm_) was performed every hour for 24 h at 26°C with 10 sec of shaking prior to each measurement. Two experiments were repeated independently.

### Inoculation of flowering apple trees in the greenhouse.

3-year-old GD trees were potted and kept in a quarantine greenhouse (closed compartment) until flowering. Selected strains were applied on each single flower using a hand sprayer (200 μl per hub) and an OD_600nm_ = 1 bacterial suspension (see next paragraph) that was 10^−2^-fold diluted in PBS buffer. Three apple trees per strain were placed in a greenhouse cabin with 60% humidity after spray inoculation. The temperature was set to 18°C (10 h) at night and 24°C (10 h) at day and 2 h ramp time. After 6 days, flower necrosis symptoms were evaluated, indicated by a darkening of tissue of the flower calyx (brownish to black: necrosis positive, green: necrosis negative).

To estimate the CFU of bacteria in spray inoculated flowers, flower petals, pedestals, and stems were removed. The remains of the apple blossom were shaken in 1 mL PBS buffer for 30 min at 1400 rpm and then vortexed for 30 sec. A serial dilution of the resulting supernatants was performed up to a 10^−7^-fold dilution. Three μl of each dilution step from each sample were transferred onto TSB agar plates containing appropriate antibiotics using a 96-replicate plater (Fig. S6). As a control, defined EA suspensions were equally diluted and spotted alongside the samples. CFU counts were performed after 24 h under a binocular microscope. For resulting supernatants that led to no cultivated colonies on the TSB agar plates, the minimal detection limit of one colony was calculated.

### Detached flower assay, visual grading.

The detached flower assay (36) was performed as previously described (37). Freshly opened flowers of 2-year-old potted GD trees were used. In autoclaved Eppendorf racks every second well of every second row was filled with two mL autoclaved tap water and sealed with office tape. Holes were punched through the tape using sterile pipette tips and finally the stems of detached flowers were inserted into the holes.

All used EA strains were grown overnight on TSB agar plates. Bacterial biomass was resuspended in PBS buffer to an OD_600nm_ = 1. Twenty μL of each bacterial suspension was added to 980 μL PBS buffer. Twenty μL of the dilution were then directly pipetted onto the hypanthium of individual flowers. Control treatments were performed using PBS buffer only.

For the three strains EA^S^, EAdfoA and EAfoxR, a total of 2 × 24 flowers were tested in two independent experiments. After inoculation of the flowers, the Eppendorf racks were transferred in a storage box laid out with water-soaked paper towels to ensure high relative humidity. Incubation was performed at 26°C for four to 5 days. After incubation each flower was visually graded based on the following scale: grade 1: calyx green; grade 2: calyx necrotic (brownish), pedicel green; grade 3: calyx and pedicel necrotic (Fig. S7). The results of both experiments were added up and the severity grade of the infection was defined as previously described (38).

### Growth on stigma and hypanthium.

To estimate the CFU of parental strain EA^S^ and mutant strains on the stigma and hypanthium of detached flowers, the OD_600nm_ = 1 (see above) bacterial suspensions were 10^−4^-fold diluted and 1.5 mL reaction tubes filled up to the rim with these suspensions. The petals and stamens of GD flowers were removed with sterile scissors and the exposed stigmata were inoculated by carefully dipping them into the bacterial suspension. Immediately after drying of the bacterial suspension, the CFU of bacteria on stigma and hypanthium of single flowers were determined (time = 0). Stigmata of each flower were cut with sterile scissors and collected in a 1.5 mL reaction tube filled with 200 μL PBS buffer. The remains of the flowers were similarly treated. For CFU determination after prolonged incubation (24 h and 72 h), flower parts were resuspended in 1 mL PBS buffer. The CFU determination was performed as described above in section “Inoculation of flowering apple trees in the greenhouse.”

### FCM (Flow cytometer) analysis.

For *in vitro* measurements of GFP expressing bacteria, a TSB overnight culture of the strains containing the appropriate antibiotics was diluted 1:100 and cultivated (240 rpm) at 26°C for 48 h. Afterwards, 1 mL of the bacterial suspension was centrifuged (30 sec, max. speed, Eppendorf MiniSpin) and the pellet washed in sterile filtered PBS.

After an additional centrifugation step the pellet was resuspended in 100 μl PBS-buffered 4% paraformaldehyde and stored at 4°C in the dark before processing.

For GFP measurement *in planta*, bacteria were cultivated overnight and bacterial suspensions adjusted to OD_600nm_ = 1 in PBS buffer were diluted 10^−5^-fold. The stamens of detached flowers were removed with sterile scissors and the exposed stigmata inoculated with 2 μL of the diluted suspensions. Inoculated flower stigmata were incubated at 26°C for 24 und 48 h. After incubation the stigmata of each flower were cut with sterile scissors and collected in a 1.5 mL reaction tube containing 100 μL sterile filtered PBS. Samples were shaken at RT for 30 min at 1400 rpm. Subsequently, the tubes were vortexed vigorously for 2 min. Ninety μL of the suspension were carefully transferred by slowly pipetting into 1.5 mL reaction tubes with 90 μL PBS-buffered 4% paraformaldehyde (Alfa Aesar). Samples were kept at 4°C in the dark for up to 24 or 48 h before the FCM analysis was performed.

Flow cytometric measurements were performed with the CytoSense benchtop FCM (Cytobuoy, Netherlands). The FCM is equipped with a 125 mV laser at a wavelength of 488 nm. The green fluorescence of GFP was detected in a range from 509 to 540 nm. Each sample was diluted with sterile PBS until a particle count between 500 and 5’000 particles per second was present during measurement (for ca. 20000 cells per flower sample). To distinguish between impurities and cells of interest a gate (subset of all measured events) was established based on forward scatter (FWS) and sidewards scatter (SWS) signals. As a control for gate selection Image In Flow (IFF) pictures were taken into account. Based on the gate the fluorescence intensity of the GFP (Fl green signal integral) was used to differentiate between GFP positive and GFP negative cells.

### Competition studies using GD orchard or greenhouse flowers.

GD flowers were collected in the apple orchard-P23 at the Agroscope Research Station in Wädenswil, Switzerland (GPS: 47°13′18.1″ N, 8°40′38.9″ E). To ensure sufficient time for the development of a microbiome in the flowers, the collected flowers had their petals open and partially brownish anthers (Fig. S8). Flowers collected in the balloon state and thus devoid of a microbiome were incubated at 26°C for several hours to trigger petal opening and allow subsequent inoculation. For pre-inoculation of flowers with the competitor P. vagans C9-1W (PW), GD flowers from the greenhouse were used (Fig. S8).

All inoculations for orchard flowers were performed by pipetting 2 μl of a 10^−5^-fold diluted OD_600nm_ = 1 suspension onto the stigmata. Initial CFU densities of the inoculum were determined by mixing 10 μl of the diluted suspension with 200 μL PBS and plating 42 μL on TSB agar plates. The plates were incubated at 26°C and CFU were counted after incubation. The inoculum was in the range of 20 to < 70 colonies.

For pre-inoculation of flowers with PW, a bacterial suspension of the strain with an OD_600nm_ = 1 was 10^−3^-fold diluted and 1.5 mL reaction tubes were filled up to the rim with the suspension. Stigmata of freshly opened flowers from GD trees grown in the greenhouse were inoculated by carefully dipping the stigmata into the suspension. On these flowers 2 μl of a 10^−4^-fold diluted bacterial suspension of the selected E. amylovora strains were pipetted onto the pre-inoculated stigmata. For CFU determination of the inoculum, the 10^−4^-fold diluted bacterial suspensions were log diluted to a 10^−5^-fold dilution. Incubation and CFU determination was performed as described above. For assessing the microorganisms colonizing the orchard-P23 flowers, balloon flowers, and greenhouse flowers, in addition to the inoculated strains, dilutions of the reisolated bacterial suspensions were also plated on TSB agar plates supplemented with rifampicin (selection for fungi) and KB agar plates containing the fungicide cycloheximide (selection for bacteria) (Fig. S3).

### DNA extraction.

Bacterial DNA from apple flowers was extracted by transferring 0.5 mL of the 1 mL flower resuspension to sterile 1.5 mL reaction tubes followed by centrifugation for 1 min at 12.100 × *g* in an Eppendorf mini centrifuge. The pellet was stored at −20°C until DNA was extracted using the BioSprint 96 DNA Plant kit (Qiagen) as described previously (39).

### Real-time PCR to determine bacterial titer in flowers.

Real-time PCRs were performed according to Pirc et al. (40) in a LightCycler 480 (Roche) using the TaqMan Universal master mix (Applied Biosystems) and the target gene *ams*C. The final reaction volume (10 μl) contained 0.9 μl of 10 μM primer Ams116F (5′-TCCCACATACTGTGAATCATCCA-3′), 0.9 μl of 10 μM primer Ams189R (5′-GGGTATTTGCGCTAATTTTATTCG-3′), 0.2 μl of 10 μM Ams141T (5′-FAM-CCA GAA TCT GGC CCG CGT ATA CCG-TAMRA-3′), 1 μl ddH_2_O, 5 μl of 2× TaqMan universal master mix (Applied Biosystems), and 2 μl of template DNA. All PCRs were conducted in triplicate and negative controls were included. The baseline was set automatically, cycling conditions were: 2 min at 50°C, 10 min at 95°C, 40 cycles of 15 s at 95°C and 1 min at 60°C. A crossing point (Cp) value above 38 would have been considered negative.

## References

[1] Bagg A, Neilands JB. 1987. Molecular mechanism of regulation of siderophore-mediated iron assimilation. Microbiol Rev 51:509–518. 10.1128/mr.51.4.509-518.1987. 2963952 PMC373130

[2] Andrews SC, Robinson AK, Rodríguez-Quiñones F. 2003. Bacterial iron homeostasis. FEMS Microbiol Rev 27:215–237. 10.1016/S0168-6445(03)00055-X. 12829269

[3] Krewulak KD, Vogel HJ. 2008. Structural biology of bacterial iron uptake. Biochim Biophys Acta 1778:1781–1804. 10.1016/j.bbamem.2007.07.026. 17916327

[4] Escolar L, Pérez-Martín J, de Lorenzo V. 1999. Opening the iron box: transcriptional metalloregulation by the Fur protein. J Bacteriol 181:6223–6229. 10.1128/JB.181.20.6223-6229.1999. 10515908 PMC103753

[5] Bagg A, Neilands JB. 1987. Ferric uptake regulation protein acts as a repressor, employing iron (II) as a cofactor to bind the operator of an iron transport operon in *Escherichia coli*. Biochemistry 26:5471–5477. 10.1021/bi00391a039. 2823881

[6] Vanneste JL. 2000. Fire Blight: the Disease and Its Causative Agent, Erwinia amylovora. CABI.

[7] Johnson KB, Stockwell VO. 1998. Management of fire blight: a case study in microbial ecology. Annu Rev Phytopathol 36:227–248. 10.1146/annurev.phyto.36.1.227. 15012499

[8] Salomone-Stagni M, Bartho JD, Polsinelli I, Bellini D, Walsh MA, Demitri N, Benini S. 2018. A complete structural characterization of the desferrioxamine E biosynthetic pathway from the fire blight pathogen *Erwinia amylovora*. J Struct Biol 202:236–249. 10.1016/j.jsb.2018.02.002. 29428557

[9] Feistner GJ, Stahl DC, Gabrik AH. 1993. Proferrioxamine siderophores of *Erwinia amylovora*. A capillary liquid chromatographic/electrospray tandem mass spectrometric study. Org Mass Spectrom 28:163–175. 10.1002/oms.1210280307.

[10] Kachadourian R, Dellagi A, Laurent J, Bricard L, Kunesch G, Expert D. 1996. Desferrioxamine-dependent iron transport in *Erwinia amylovora* CFBP1430: cloning of the gene encoding the ferrioxamine receptor FoxR. Biometals 9:143–150. 10.1007/BF00144619. 8744897

[11] Smits THM, Duffy B. 2011. Genomics of iron acquisition in the plant pathogen *Erwinia amylovora*: insights in the biosynthetic pathway of the siderophore desferrioxamine E. Arch Microbiol 193:693–699. 10.1007/s00203-011-0739-0. 21814817

[12] Dellagi A, Brisset MN, Paulin JP, Expert D. 1998. Dual role of desferrioxamine in *Erwinia amylovora* pathogenicity. Mol Plant Microbe Interact 11:734–742. 10.1094/MPMI.1998.11.8.734. 9675889

[13] Santos Kron A, Zengerer V, Bieri M, Dreyfuss V, Sostizzo T, Schmid M, Lutz M, Remus-Emsermann MNP, Pelludat C. 2020. *Pseudomonas orientalis* F9 pyoverdine, safracin, and phenazine mutants remain effective antagonists against *Erwinia amylovora* in apple flowers. Appl Environ Microbiol 86:e02620-19. 10.1128/AEM.02620-19. 32033956 PMC7117935

[14] Remus-Emsermann MNP, Kowalchuk GA, Leveau JHJ. 2013. Single-cell versus population-level reproductive success of bacterial immigrants to pre-colonized leaf surfaces. Environ Microbiol Rep 5:387–392. 10.1111/1758-2229.12040. 23754719

[15] Neilands JB. 1995. Siderophores: structure and function of microbial iron transport compounds. J Biol Chem 270:26723–26726. 10.1074/jbc.270.45.26723. 7592901

[16] Pelludat C, Hogardt M, Heesemann J. 2002. Transfer of the core region genes of the *Yersinia enterocolitica* WA-C serotype O:8 high-pathogenicity island to *Y. enterocolitica* MRS40, a strain with low levels of pathogenicity, confers a yersiniabactin biosynthesis phenotype and enhanced mouse virulence. Infect Immun 70:1832–1841. 10.1128/IAI.70.4.1832-1841.2002. 11895945 PMC127873

[17] Fetherston JD, Kirillina O, Bobrov AG, Paulley JT, Perry RD. 2010. The yersiniabactin transport system is critical for the pathogenesis of bubonic and pneumonic plague. Infect Immun 78:2045–2052. 10.1128/IAI.01236-09. 20160020 PMC2863531

[18] Crouch M-LV, Castor M, Karlinsey JE, Kalhorn T, Fang FC. 2008. Biosynthesis and IroC-dependent export of the siderophore salmochelin are essential for virulence of *Salmonella enterica* serovar Typhimurium. Mol Microbiol 67:971–983. 10.1111/j.1365-2958.2007.06089.x. 18194158

[19] Franza T, Mahé B, Expert D. 2005. *Erwinia chrysanthemi* requires a second iron transport route dependent of the siderophore achromobactin for extracellular growth and plant infection. Mol Microbiol 55:261–275. 10.1111/j.1365-2958.2004.04383.x. 15612933

[20] Taguchi F, Suzuki T, Inagaki Y, Toyoda K, Shiraishi T, Ichinose Y. 2010. The siderophore pyoverdine of *Pseudomonas syringae* pv. *tabaci* 6605 is an intrinsic virulence factor in host tobacco infection. J Bacteriol 192:117–126. 10.1128/JB.00689-09. 19854904 PMC2798240

[21] Temple TN, Stockwell VO, Loper JE, Johnson KB. 2004. Bioavailability of iron to *Pseudomonas fluorescens* strain A506 on flowers of pear and apple. Phytopathology 94:1286–1294. 10.1094/PHYTO.2004.94.12.1286. 18943698

[22] Smits THM, Rezzonico F, Kamber T, Blom J, Goesmann A, Frey JE, Duffy B. 2010. Complete genome sequence of the fire blight pathogen *Erwinia amylovora* CFBP 1430 and comparison to other *Erwinia* spp. Mol Plant Microbe Interact 23:384–393. 10.1094/MPMI-23-4-0384. 20192826

[23] Franza T, Expert D. 2013. Role of iron homeostasis in the virulence of phytopathogenic bacteria: an “à la carte” menu. Mol Plant Pathol 14:429–438. 10.1111/mpp.12007. 23171271 PMC6638640

[24] Janakiraman A, Slauch JM. 2000. The putative iron transport system SitABCD encoded on SPI1 is required for full virulence of *Salmonella typhimurium*. Mol Microbiol 35:1146–1155. 10.1046/j.1365-2958.2000.01783.x. 10712695

[25] Bearden SW, Staggs TM, Perry RD. 1998. An ABC transporter system of *Yersinia pestis* allows utilization of chelated iron by *Escherichia coli* SAB11. J Bacteriol 180:1135–1147. 10.1128/JB.180.5.1135-1147.1998. 9495751 PMC107000

[26] Bearden SW, Perry RD. 1999. The Yfe system of *Yersinia pestis* transports iron and manganese and is required for full virulence of plague. Mol Microbiol 32:403–414. 10.1046/j.1365-2958.1999.01360.x. 10231495

[27] Cao J, Woodhall MR, Alvarez J, Cartron ML, Andrews SC. 2007. EfeUOB (YcdNOB) is a tripartite, acid-induced and CpxAR-regulated, low-pH Fe2+ transporter that is cryptic in *Escherichia coli* K-12 but functional in *E. coli* O157:H7. Mol Microbiol 65:857–875. 10.1111/j.1365-2958.2007.05977.x. 17627767

[28] Dellagi A, Reis D, Vian B, Expert D. 1999. Expression of the ferrioxamine receptor gene of *Erwinia amylovora* CFBP 1430 during pathogenesis. Mol Plant Microbe Interact 12:463–466. 10.1094/MPMI.1999.12.5.463. 10226380

[29] King EO, Ward MK, Raney DE. 1954. Two simple media for the demonstration of pyocyanin and fluorescin. J Lab Clin Med 44:301–307. 13184240

[30] Galán JE, Ginocchio C, Costeas P. 1992. Molecular and functional characterization of the *Salmonella* invasion gene *invA*: homology of InvA to members of a new protein family. J Bacteriol 174:4338–4349. 10.1128/jb.174.13.4338-4349.1992. 1624429 PMC206218

[31] Skorupski K, Taylor RK. 1996. Positive selection vectors for allelic exchange. Gene 169:47–52. 10.1016/0378-1119(95)00793-8. 8635748

[32] Miller VL, Mekalanos JJ. 1988. A novel suicide vector and its use in construction of insertion mutations: osmoregulation of outer membrane proteins and virulence determinants in *Vibrio cholerae* requires toxR. J Bacteriol 170:2575–2583. 10.1128/jb.170.6.2575-2583.1988. 2836362 PMC211174

[33] Simon R, Priefer U, Pühler A. 1983. A broad host range mobilization system for in vivo genetic engineering: transposon mutagenesis in Gram negative bacteria. Nat Biotechnol 1:784–791. 10.1038/nbt1183-784.

[34] Stecher B, Hapfelmeier S, Müller C, Kremer M, Stallmach T, Hardt W-D. 2004. Flagella and chemotaxis are required for efficient induction of *Salmonella enterica* serovar Typhimurium colitis in streptomycin-pretreated mice. Infect Immun 72:4138–4150. 10.1128/IAI.72.7.4138-4150.2004. 15213159 PMC427403

[35] Schwyn B, Neilands JB. 1987. Universal chemical assay for the detection and determination of siderophores. Anal Biochem 160:47–56. 10.1016/0003-2697(87)90612-9. 2952030

[36] Pusey PL. 1997. Crab apple blossoms as a model for research on biological control of fire blight. Phytopathology 87:1096–1102. 10.1094/PHYTO.1997.87.11.1096. 18945005

[37] Zengerer V, Schmid M, Bieri M, Müller DC, Remus-Emsermann MNP, Ahrens CH, Pelludat C. 2018. F9: a potent antagonist against phytopathogens with phytotoxic effect in the apple flower. Front Microbiol 9:145. 10.3389/fmicb.2018.00145. 29479340 PMC5811506

[38] Llop P, Cabrefiga J, Smits THM, Dreo T, Barbé S, Pulawska J, Bultreys A, Blom J, Duffy B, Montesinos E, López MM. 2011. *Erwinia amylovora* novel plasmid pEI70: complete sequence, biogeography, and role in aggressiveness in the fire blight phytopathogen. PLoS One 6:e28651. 10.1371/journal.pone.0028651. 22174857 PMC3235134

[39] Gschwend F, Braun-Kiewnick A, Widmer F, Pelludat C. 2021. Apple blossoms from a swiss orchard with low-input plant protection regime reveal high abundance of potential fire blight antagonists. Phytobiomes J 5:145–155. 10.1094/PBIOMES-04-20-0033-R.

[40] Pirc M, Ravnikar M, Tomlinson J, Dreo T. 2009. Improved fire blight diagnostics using quantitative real-time PCR detection of *Erwinia amylovora* chromosomal DNA. Plant Pathology 58:872–881. 10.1111/j.1365-3059.2009.02083.x.

[41] Hanahan D. 1983. Studies on transformation of *Escherichia coli* with plasmids. J Mol Biol 166:557–580. 10.1016/s0022-2836(83)80284-8. 6345791

[42] Paulin JP, Samson R. 1973. Le feu bactérien en France II. Caractères des souches *d’Erwinia amylovora* (Burill) Winslow et al. isolées du foyer franco‐belge. Ann Phytopathol 5:389–397.

[43] Smits THM, Rezzonico F, Pelludat C, Goesmann A, Frey JE, Duffy B. 2010. Genomic and phenotypic characterization of a nonpigmented variant of *Pantoea vagans* biocontrol strain C9-1 lacking the 530-kb megaplasmid pPag3. FEMS Microbiol Lett 308:48–54. 10.1111/j.1574-6968.2010.01994.x. 20487014

