# Supplementary material for: Priority Effects in the Apple Flower Determine If the Siderophore Desferrioxamine Is a Virulence Factor for Erwinia amylovora CFBP1430
Source: Appl Environ Microbiol. 2022 Mar 14;88(7):e02433-21. doi: 10.1128/aem.02433-21 (PMC9004392; doi:10.1128/aem.02433-21)
Supplement: Supplemental file 1 — Fig. S1 to S8. Download aem.02433-21-s0001.pdf, PDF file, 1.6 MB [file aem.02433-21-s0001.pdf]

Supplementary material

Priority effects in the apple flower determine if the siderophore desferrioxamine is a virulence factor for *Erwinia amylovora* CFBP1430

Laurin Müller, Denise C. Müller, Sandrine Kammerecker, Marco Fluri, Lukas Neutsch, Mitja Remus Emsermann, and Cosima Pelludat

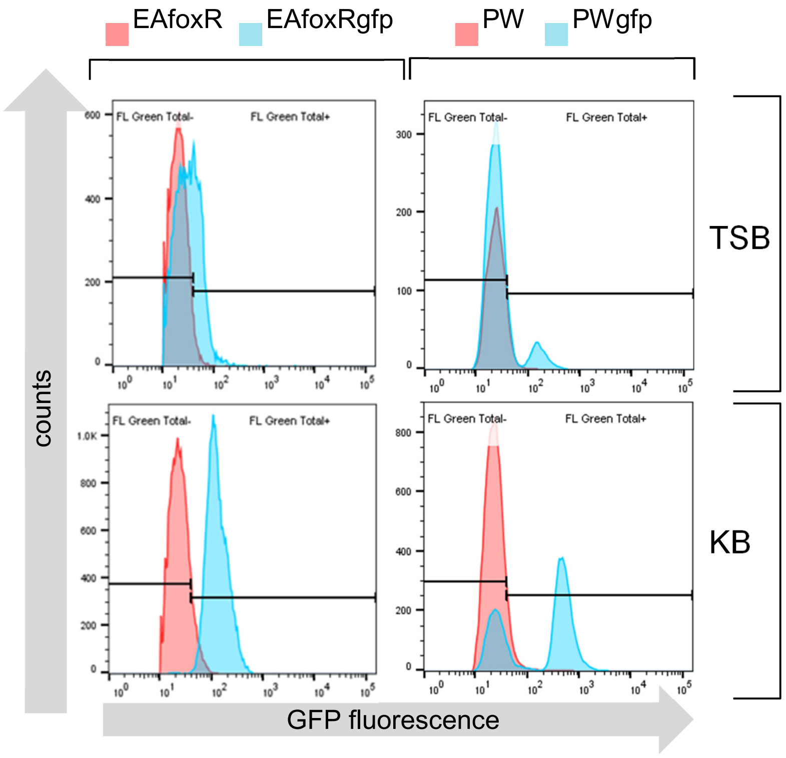

Fig S1 GFPmut2 expression under the control of the *foxR* promoter in mutant EAfoxR (EAfoxRgfp, blue) and control strain PW (PWgfp, blue). Strains were cultivated in TSB and KB medium for 48 h. Corresponding strains lacking the reporter construct (in red) were used as a negative control (EAFoxgfp in TSB: GFP positive 52%, in KB: GFP positive 93%; PWgfp in TSB: GFP positive 10%, in KB: GFP positive 61%).

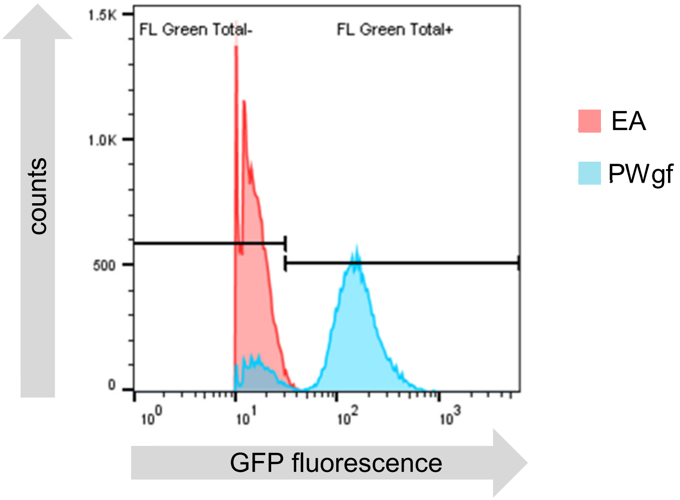

Fig S2 GFPmut2 expression in strain PWgfp (blue) under the control of the EA*foxR* promoter. *E. amylovora* CFBP1430 (red), lacking the reporter construct is the negative control (PWgfp: GFP positive 17044 out of 20127 cells, 84.7%).

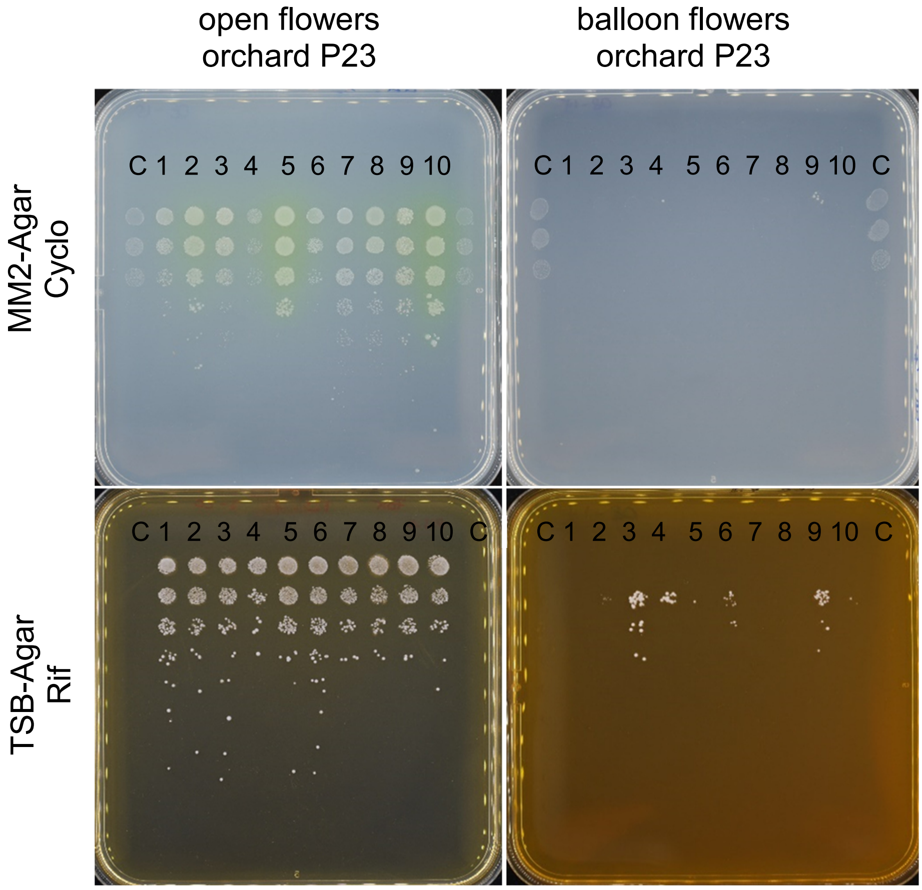

Fig S3 Microbiome in open and balloon GD flowers from orchard P23 inoculated with *E. amylovora* strains.

To detect other bacteria in the *E. amylovora*-inoculated flowers, the re-isolated bacterial suspensions (lane 1 to 10, C = *E. amylovora* dilution control) were plated additionally on MM2 agar, on which EA strains hardly grow. To prevent fungal growth, the fungicide cycloheximide (Cyclo) was added. To select for fungi, the suspensions were also plated on TSB agar containing the antibiotic rifampicin (Rif). Differences between open and balloon flowers regarding the presence/absence of a microbiome are visualized.

The suspensions of greenhouse flowers gave similar results to those of balloon flowers when plated on MM2-Cyclo and TSB-Rif plates.

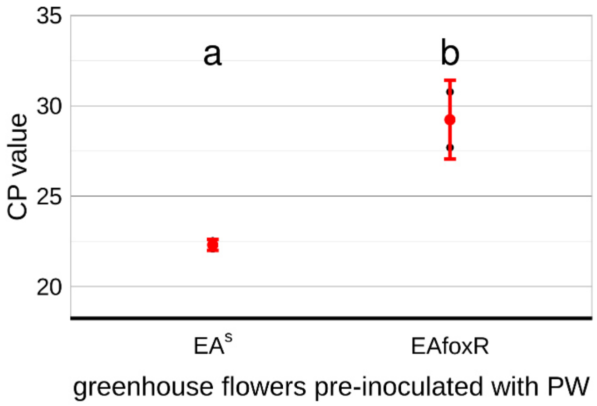

Fig S4 CP values of EA^{S} and mutant EAfoxR 48 h p.i. onto PW pre-inoculated GD flowers from the greenhouse. The DNA template was extracted from two samples. Each sample consisted of ten pooled bacterial suspensions reisolated from infected GD flowers. qPCR was performed with an *amsC* (amylovoran synthesis) specific probe. Error bars represent the standard deviation of the two means.

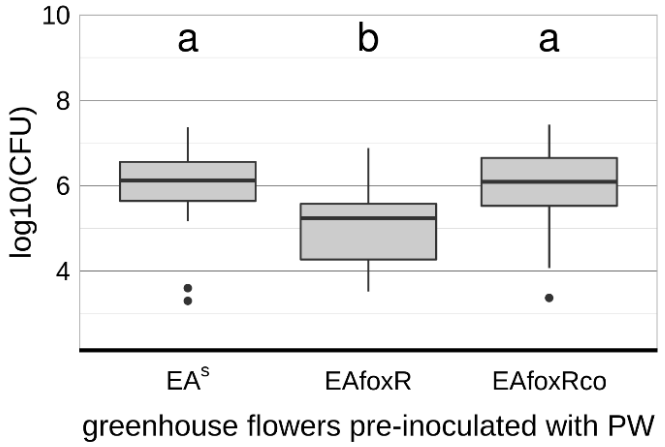

Fig S5 CFU of EA^S (n=20), mutant EAfoxR (n=19) and complemented mutant EAfoxRco (n=20) 72 h p.i. onto PW pre-inoculated GD flowers from the greenhouse. Error bars represent the standard deviation of the mean. Significant differences between treatments are marked with different letters (p-value < 0.05, one-way ANOVA, Tukey's multiple comparison test).

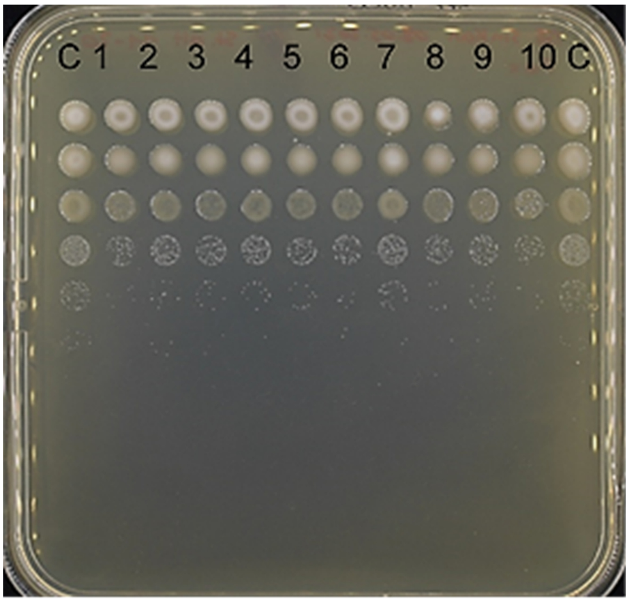

Fig S6 Determination of CFU of *E. amylovora* bacteria reisolated from GD flowers. A serial dilution of the inoculated *E. amylovora* bacteria reisolated from inoculated flowers (samples 1 to 10) was performed to a 10^{-7}-fold dilution. Three µl of each dilution level of each sample were transferred onto TSB agar plates with appropriate selection antibiotics using a 96-replicate plater. As control (C), a defined *E. amylovora* suspension was equally diluted and transferred together with the samples.

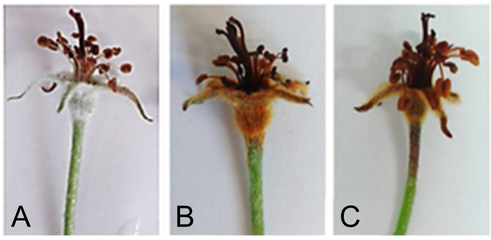

Fig S7 Visual necrosis grades of *E. amylovora* infected apple flowers.

A: grade 1: calyx green; **B:** grade 2: calyx necrotic (brownish), pedicel green; **C:** grade 3: calyx and pedicel necrotic.

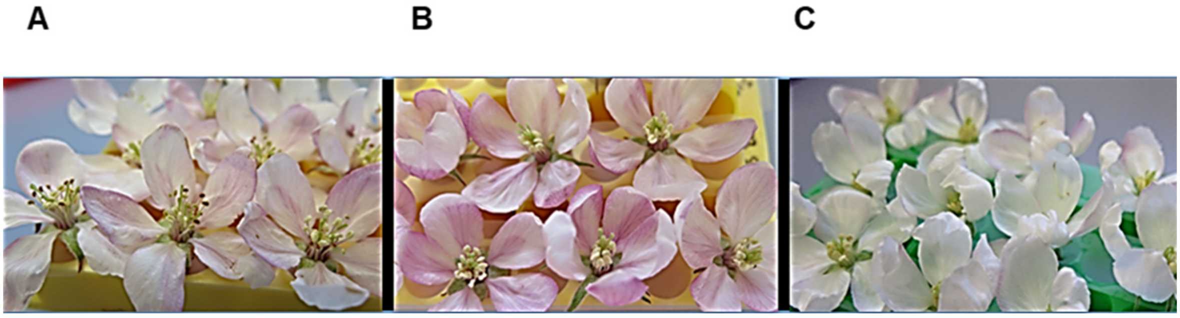

Fig S8 Selected GD flowers. **A:** open (microbiome bearing), from orchard P23, stamens partly brownish; **B:** collected in balloon stage from orchard P23, incubated at 26°C until petals opened; **C:** freshly opened, from greenhouse (semi-sterile).
